# Supplementary material for: Comparison of High Hydrostatic Pressure, Ultrasound, and Heat Treatments on the Quality of Strawberry–Apple–Lemon Juice Blend
Source: Foods. 2020 Feb 19;9(2):218. doi: 10.3390/foods9020218 (PMC7074247; doi:10.3390/foods9020218)
Supplement: Supplementary file 1 [file foods-09-00218-s001.pdf]

Supplemental table captions

Table S1 Sensory attributes and reference scores used for sensory evaluation of juice blend.

Table S1 Sensory attributes and reference scores used for sensory evaluation of juice blend.

Sensory attributes		Reference scores
Taste	Juice is moderately sweet and sour, even and delicate	30~40
	Juice is slightly sour or sweet, and a little grainy	10~30
	Juice is very sour or sweet, and very grainy	0~10
Aroma	It has a rich blended flavor of strawberry, apple and lemon; no odor	20~30
	It has some blended flavor of strawberry, apple and lemon; little odor	10~20
	It has no blended flavor of strawberry, apple and lemon; with odor	0~10
Color	The color is bright red and uniform	10~15
	The color is light or dark red, and uneven	5~10
	The color is brown-red and uneven	0~5
Consistency	No precipitation or bubbles in juice, and uniform	10~15
	Some precipitation and bubbles in juice	5~10
	Much precipitation and many bubbles in juice, and uneven	0~5
